# Artificial intelligence vs. dermoscopy for malignancy risk stratification of pigmented skin lesions: a systematic review and meta-analysis for public health

**DOI:** 10.3389/fmed.2026.1883944

**Published:** 2026-07-28

**Authors:** Ming Diao, Yaxi Zhu, Hailing Tang

**Affiliations:** Pengzhou People's Hospital, Pengzhou, Sichuan, China

**Keywords:** artificial intelligence, dermoscopy, diagnostic accuracy, melanoma, meta-analysis, pigmented skin lesions

## Abstract

**Background:**

Accurate risk stratification of pigmented skin lesions is essential for early melanoma detection and for reducing unnecessary excisions. Although artificial intelligence (AI) is increasingly applied to dermoscopic image analysis, its diagnostic performance compared to dermoscopy remains unclear.

**Objective:**

This study aimed to compare the diagnostic performance of AI, dermoscopy, and AI-assisted clinicians for malignancy risk stratification of pigmented skin lesions.

**Methods:**

PubMed, Embase, Web of Science, and the Cochrane Library were systematically searched for studies evaluating AI, dermoscopy, or AI-assisted clinicians in diagnosing pigmented or melanoma-suspected skin lesions. Diagnostic performance metrics were calculated from extracted or reconstructed data, and study quality was assessed using the Quality Assessment of Diagnostic Accuracy Studies-2 tool (QUADAS-2) and QUADAS-comparative (QUADAS-C).

**Results:**

A total of 2,571 records were identified, and 10 studies were included in the primary quantitative analysis, contributing 17 diagnostic arms. These included 10 dermoscopy arms, 6 AI-alone arms, and 1 AI-assisted clinician arm. In the dermoscopy group, sensitivity ranged from 0.418 to 0.966, and specificity ranged from 0.293 to 0.975. In the AI group, sensitivity ranged from 0.164 to 0.968, and specificity ranged from 0.374 to 0.983. AI-assisted clinicians showed a sensitivity of 1.000 and a specificity of 0.837 in the single available study. Overall, AI and dermoscopy showed overlapping diagnostic performance, although substantial variability was observed across AI algorithms and clinical settings. Deeks’ funnel plots showed no evidence of significant publication bias in either the AI group or the dermoscopy group.

**Conclusion:**

Autonomous AI showed diagnostic performance broadly comparable to dermoscopy. Although AI achieved slightly higher pooled specificity, its sensitivity was lower, indicating no clear clinical advantage over conventional dermoscopic assessment. Evidence on AI-assisted clinicians was limited to a single diagnostic arm; therefore, this finding should be interpreted as hypothesis-generating rather than as evidence to support immediate clinical implementation. Further comparative studies using standardized AI-assisted workflows, comparable physician expertise, and patient-centered outcomes are required before practical recommendations can be made.

**Systematic review registration:**

Registered with PROSPERO (CRD420261389834).

## 1 Introduction

Malignant melanoma is among the most aggressive forms of skin cancer, making early detection and timely surgical intervention crucial for improving patient outcomes. In routine dermatologic practice, pigmented skin lesions encompass a highly heterogeneous diagnostic spectrum, ranging from innocuous melanocytic nevi, through dysplastic proliferations, to potentially fatal melanomas (1–3). Consequently, accurate malignancy risk stratification is a central clinical imperative. Clinicians face the dual challenge of maximizing diagnostic sensitivity to avoid the catastrophic consequences of missed melanomas while simultaneously maintaining sufficient specificity to reduce the physical morbidity and economic burden associated with the unnecessary excision of benign lesions with melanoma-like features (4, 5).

To navigate this morphological complexity, dermoscopy, a non-invasive diagnostic technique, has become an indispensable tool in dermatology by enabling improved visualization of subsurface skin structures. Compared with unaided visual inspection, it substantially increases diagnostic accuracy for melanoma and helps reduce unnecessary excisions of benign lesions (6, 7). Despite being the current standard of care, its diagnostic performance remains highly dependent on the clinician’s level of experience. Pattern recognition and heuristic evaluations vary significantly; thus, the diagnostic yield achieved by an expert dermoscopist frequently exceeds that of a general practitioner or a dermatologist with limited optical training (8, 9).

In recent years, artificial intelligence (AI) systems have shown promising results in the automated analysis and classification of dermoscopic images. Convolutional neural networks (CNNs), in particular, have become the most commonly used approach in this field and form the basis for most modern algorithms for classifying skin lesions. Numerous retrospective studies have reported diagnostic performances comparable to, or even exceeding, those of expert dermatologists (10, 11). Nevertheless, the majority of studies evaluating AI performance have been retrospective and have relied on curated image datasets that may not reflect the complexity of everyday clinical practice. These retrospective analyses are limited in their ability to assess generalizability, risk of bias, and real-world diagnostic effects, frequently overestimating an algorithm’s translational accuracy when confronted with patients with diverse skin tones or unrepresented lesion morphologies (12, 13).

Historically, the discourse surrounding machine learning in dermatology has focused on an adversarial narrative, primarily emphasizing direct comparisons between isolated AI algorithms and clinicians (14). Considerably limited attention has been paid to the pragmatic integration of AI as a synergistic tool, specifically AI-assisted clinicians designed to augment human decision-making, or as a benchmarked alternative to standalone dermoscopy, a mature non-invasive diagnostic technique (15). While individual studies have shown promising results for AI in skin cancer diagnosis, there remains a lack of systematic evidence directly comparing AI alone, standalone dermoscopy, and AI-assisted clinicians in prospective clinical settings (16). By synthesizing available evidence, this systematic review and meta-analysis aimed to compare the diagnostic performance of AI alone, standalone dermoscopy, and AI-assisted clinicians for malignancy risk stratification of pigmented skin lesions, ultimately informing the current state of clinical readiness for these technologies in real-world settings.

## 2 Methods

The systematic review and meta-analysis adhered to the guidelines outlined in the Preferred Reporting Items for Systematic Reviews and Meta-Analyses (PRISMA) statement. The study was registered in the International Prospective Register of Systematic Reviews (PROSPERO) (CRD420261389834).

### 2.1 Eligibility criteria

We included diagnostic accuracy studies that evaluated AI, dermoscopy, or AI-assisted clinicians for malignancy risk stratification of pigmented skin lesions. Eligible studies included patients with pigmented, melanocytic, or melanoma-suspected skin lesions and reported diagnostic performance data for at least one of the following diagnostic approaches: AI alone, dermoscopy or dermatologist-performed dermoscopic assessment, or dermatologists assisted by AI. Studies were eligible if the reference standard was histopathological examination or histopathology combined with clinical follow-up or expert consensus for non-excised benign lesions. Studies were required to provide sufficient quantitative data to extract or calculate true-positive (TP), false-positive (FP), false-negative (FN), and true-negative (TN) values, or to report sensitivity and specificity from which a 2 × 2 diagnostic table could be converted into.

The primary analysis focused on prospective clinical studies or prospective diagnostic validation studies using dermoscopic images or dermoscopy-based assessment. Diagnostic arms were categorized into three groups: AI alone, dermoscopy, and dermatologists assisted by AI. Studies evaluating non-dermoscopic AI applications, mobile applications, reader studies, or non-AI imaging techniques were considered for supplementary analysis when relevant but were not combined with the main pooled analysis.

We excluded studies based on the following criteria: (1) reviews, editorials, commentaries, letters, conference abstracts, or protocols; (2) purely algorithm development studies without clinical validation; (3) studies using only public retrospective image datasets without a clinical diagnostic setting; (4) studies not involving pigmented skin lesions, melanocytic lesions, melanoma, or skin cancer risk stratification; (5) studies without an appropriate reference standard; (6) studies with insufficient data to extract or calculate diagnostic accuracy outcomes; or (7) duplicated study populations, in which case the most complete or most clinically relevant report was retained. Emerging non-invasive or visual enhancement technologies, including spectrum-aided visual enhancement, hyperspectral or multispectral imaging, optical coherence tomography, and reflectance confocal microscopy, were considered as contextual evidence when relevant to melanoma risk assessment. However, they were not included in the primary quantitative synthesis unless they met the predefined criteria for prospective clinical diagnostic validation, evaluated a comparable diagnostic decision threshold, and provided sufficient 2 × 2 diagnostic accuracy data. Studies reporting object detection or image enhancement outcomes, such as precision, recall, F1-score, or mean average precision without extractable TP, FP, FN, and TN values, were therefore summarized narratively rather than included in the pooled analysis.

### 2.2 Search strategy and study selection

A systematic literature search was conducted in PubMed, Embase, Web of Science, and the Cochrane Library from database inception to 31 January 2026. The search strategy combined controlled vocabulary terms and free-text terms related to artificial intelligence, machine learning, dermoscopy, pigmented skin lesions, melanoma, and diagnostic accuracy. No restrictions on publication year or language were applied at the electronic search stage. Animal-only studies were excluded where database syntax allowed, and non-human studies were further excluded during screening. Publication-type filters were not used during database searching; reviews, editorials, commentaries, letters, conference abstracts, protocols, and algorithm-development-only studies lacking clinical diagnostic validation were excluded during eligibility assessment.

The complete database-specific search strings, search fields, and filters are provided in Supplementary Table S2. Briefly, the main search combined four concept blocks: (1) artificial intelligence or machine learning; (2) dermoscopy or dermatoscopy; (3) melanoma, pigmented skin lesions, melanocytic lesions, or skin cancer; and (4) diagnostic accuracy, sensitivity, specificity, ROC, area under the curve (AUC), diagnostic performance, or risk stratification. Terms related to skin tone, skin of color, Fitzpatrick type, and algorithmic fairness were not used as mandatory terms in the main search because doing so could have restricted retrieval to studies explicitly mentioning these concepts and thereby reduced search sensitivity. Instead, these terms were used in a supplementary targeted search to inform the narrative discussion of dataset representativeness, skin tone diversity, and algorithmic fairness.

In addition to electronic database searching, the reference lists of all included studies and relevant systematic reviews were manually screened to identify potentially eligible studies not captured by the database search. Forward citation tracking was also performed for key included studies where appropriate. All records were imported into EndNote X7 for deduplication. Two reviewers independently screened titles and abstracts, followed by full-text assessment according to predefined eligibility criteria. Disagreements were resolved by discussion or consultation with a third reviewer. Reasons for full-text exclusion were recorded and summarized in the PRISMA flow diagram.

### 2.3 Data extraction and quality assessment

Data were extracted using a standardized data extraction form. The extracted information included the first author, publication year, country or region, study design, sample size, number of patients and lesions, and the number of disease-positive and disease-negative lesions according to the binary target condition used for each analysis, diagnostic approach, AI system or dermoscopy method, comparator, reference standard, and follow-up method when applicable. For each eligible diagnostic arm, TP, FP, FN, and TN values were extracted directly when available. When a 2 × 2 table was not explicitly reported, the values were recalculated from reported sensitivity, specificity, and the number of malignant and non-malignant lesions. For multi-class AI classifiers, predicted malignant categories, including melanoma and other malignant non-melanoma categories where available, or management outputs recommending biopsy or excision, were coded as test-positive. Predicted benign categories or non-biopsy/non-excision outputs were coded as test-negative.

Diagnostic arms were classified into three predefined groups: AI alone, dermoscopy, and AI-assisted clinicians. When a study reported multiple diagnostic arms based on the same study population, all eligible arms were extracted separately for descriptive presentation and for the primary arm-level synthesis, as different algorithms, reader groups, diagnostic thresholds, or clinical workflows represented distinct index test conditions. However, to reduce the potential influence of correlated arms and double counting of the same participants or lesions, we prespecified sensitivity analyses using a restricted dataset.

For the restricted sensitivity analyses, one diagnostic arm was retained per study within each diagnostic category whenever multiple arms originated from the same population. The retained arm was selected according to the following hierarchy: first, the diagnostic arm prespecified as the primary index test or primary comparator in the original study; second, the arm most consistent with routine clinical practice; third, the arm using the authors’ recommended or clinically intended operating threshold; fourth, the arm with the most complete 2 × 2 diagnostic data; and fifth, for dermoscopy arms, expert or standard-of-care dermoscopic assessment was prioritized over novice or experimental reader groups. For AI-alone arms, locked or clinically deployed algorithms and predefined thresholds were prioritized over exploratory or alternative algorithmic outputs. For AI-assisted clinician assessment, the arm was retained only when clinicians made decisions with explicit access to AI output in the same clinical population.

All extracted diagnostic arms and reconstructed 2 × 2 tables are presented in Supplementary Table S3. Arm-level inclusion and exclusion decisions for the one-arm-per-study sensitivity analysis are reported in Supplementary Table S4. The methodological quality of the included studies was assessed using the Quality Assessment of Diagnostic Accuracy Studies 2 tool (QUADAS-2) (17). Four domains were evaluated: patient selection, index test, reference standard, and flow and timing. Each domain was judged as low, high, or unclear risk of bias. Applicability concerns were assessed for patient selection, index test, and reference standard. For studies directly comparing AI with dermoscopy or dermatologist assessment within the same clinical setting, the QUADAS-C tool (18) was additionally used to evaluate the risk of bias in comparative diagnostic accuracy.

### 2.4 Statistical analysis

All analyses were performed using R version 4.3.0. For each diagnostic arm, TP, FP, FN, and TN values were extracted or reconstructed from reported sensitivity, specificity, and the number of disease-positive and disease-negative lesions. Reconstructed counts were rounded to the nearest integer and checked to ensure consistency with the reported denominators. Sensitivity, specificity, accuracy, and balanced accuracy were calculated for each arm. Study-level sensitivity and specificity were presented with 95% confidence intervals.

For pooling, sensitivity and specificity were transformed to the logit scale before random-effects meta-analysis. When a 2 × 2 table contained a zero cell, a continuity correction of 0.5 was added to all four cells before logit transformation. Pooled estimates were back-transformed and reported as proportions with 95% confidence intervals. Where feasible, bivariate random-effects models were used to jointly estimate sensitivity and specificity. Because several studies contributed multiple arms, the primary arm-level analysis was supplemented by a one-arm-per-study sensitivity analysis according to the prespecified hierarchy described in Section 2.3. Deeks’ funnel plots were used to assess small-study effects when appropriate (19).

## 3 Results

### 3.1 Study selection

**Figure 1:**
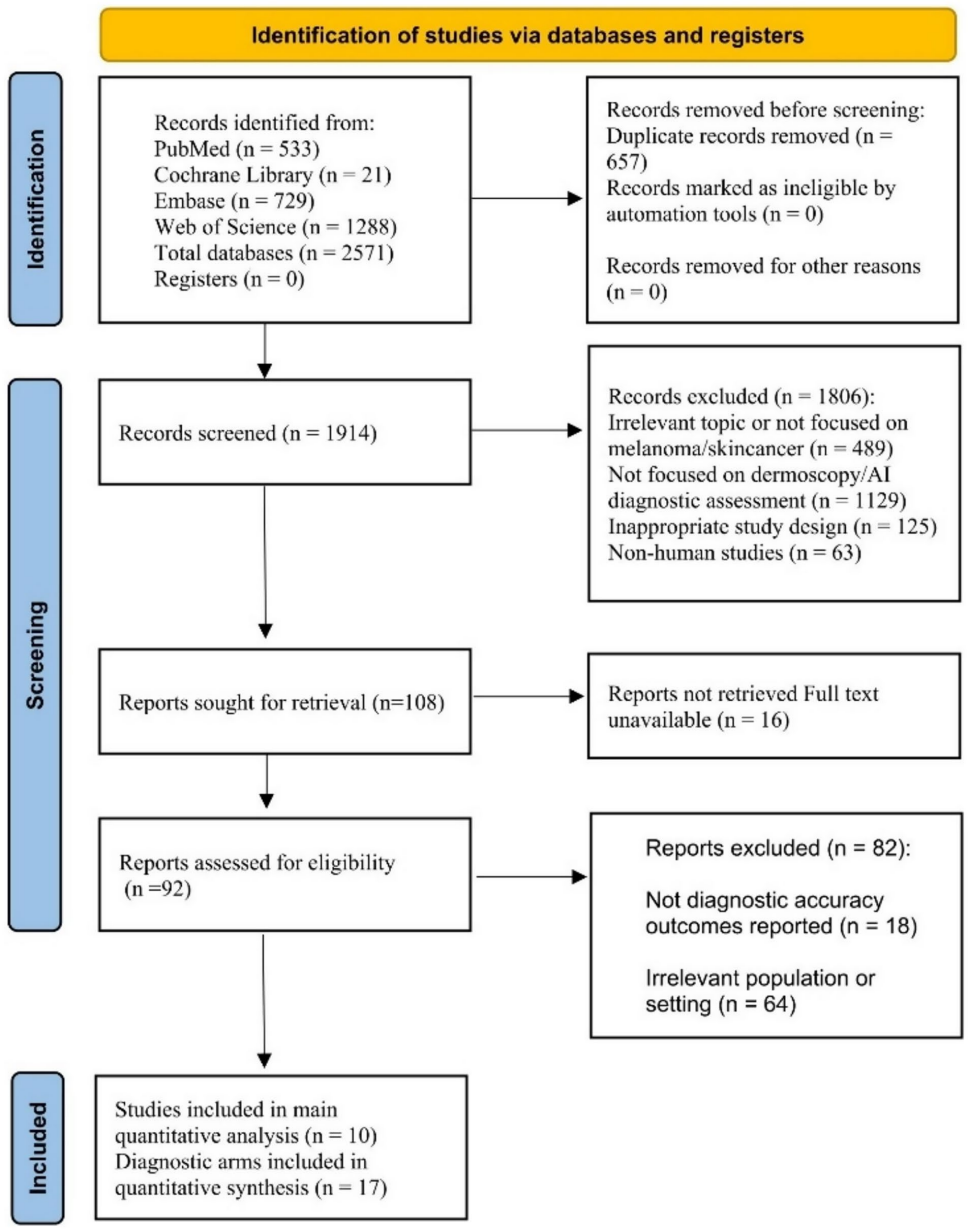
PRISMA flow diagram of study selection. A total of 2,571 records were identified from the PubMed, Cochrane Library, Embase, and Web of Science databases. After removing 657 duplicates, 1,914 records were screened. Finally, 10 studies were included in the main quantitative analysis.

A total of 2,571 records were identified through database searching. After removing 657 duplicates, 1,914 records were screened by title and abstract. Among them, 1,806 records were excluded as irrelevant. Subsequently, 92 full-text articles were assessed for eligibility. Finally, 10 studies contributing 17 diagnostic arms were included in the primary diagnostic performance analysis. The 17 diagnostic arms consisted of 10 dermoscopy arms, 6 AI-alone arms, and 1 AI-assisted clinician arm. The study selection process is shown in Figure 1.

### 3.2 Study characteristics

A total of 10 studies published between 2002 and 2024 were included in the main quantitative analysis (16, 20–28). These studies were conducted in Italy, Austria, Germany, Canada, the United Kingdom, the United States, and Australia. The majority of studies were prospective diagnostic accuracy studies or prospective clinical validation studies, enrolling patients with clinically suspicious pigmented, melanocytic, or melanoma-suspected lesions.

Sample sizes varied across studies, with the number of lesions ranging from 125 to 1,910. Histopathology was the main reference standard, while clinical follow-up or expert consensus was used for some non-excised benign lesions. Diagnostic approaches were classified into three groups: dermoscopy, AI alone, and AI-assisted clinicians. The dermoscopy group included dermatologist or clinician assessment based on dermoscopy; the AI group included systems such as FotoFinder, All Data Are Ext (ADAE), AI 7-class, AI ISIC, and Moleanalyzer Pro CNN; and only one study evaluated dermatologists assisted by AI. The characteristics of the included studies are shown in Table 1.

**Table 1:** Characteristics of the included studies.

Study	Country	Study design	Patients/lesions	Disease-positive/disease-negative lesions	Index test/AI system	Comparator	Reference standard	Included analysis group
Bono (20)	Italy	Prospective diagnostic accuracy study	298 patients/313 lesions	66/247	Dermoscopy; telespectrophotometry	Clinical naked-eye examination	Histopathology for all lesions	Dermoscopy
Dreiseitl (21)	Austria	Prospective clinical trial	511 patients/3,021 lesion examinations available for analysis	27/431	Neural network-based decision support system; expert dermoscopy	Expert dermatologist using dermoscopy	Histopathology for suspected lesions; follow-up for others	Dermoscopy; AI supplementary analysis
Heinlein (22)	Germany	Prospective multicenter diagnostic study	1716 patients/1910 suspicious lesions	750/1,160	ADAE open-source AI algorithm with real test-time augmentation	Dermatologists using dermoscopy	Histopathology for excised suspicious lesions	Dermoscopy; AI sensitivity analysis
Langley (23)	Canada	Prospective diagnostic accuracy study	125 patients/125 lesions	37/88	Dermoscopy; confocal scanning laser microscopy	Dermoscopy vs. CSLM	Histopathology for all lesions	Dermoscopy
MacLellan (24)	Canada	Prospective diagnostic accuracy study	184 patients/209 lesions	59/150	FotoFinder Moleanalyzer Pro; FotoFinder Tuebinger; MelaFind; Verisante Aura	Local dermatologist; teledermoscopy	Histopathology for all lesions	Dermoscopy; AI
Maier (25)	Germany	Prospective diagnostic accuracy study	195 lesions initially; 144 lesions included in the final analysis	26/118	SkinVision smartphone application using fractal image analysis	Dermatologists using clinical and dermoscopic assessment	Histopathology for all lesions	Dermoscopy; AI supplementary analysis
Marchetti (26)	USA	Prospective observational clinical validation study	435 participants/603 lesions	95/508	ADAE open-source dermoscopy-based AI algorithm	Dermatologist assessment before and after AI exposure	Histopathology for all lesions	AI
Menzies (27)	Australia; Austria	Multicenter prospective diagnostic clinical trial	Diagnostic trial: 124 patients/172 lesions	84/88 for all-malignancy diagnostic trial; 55/117 for melanoma-specific endpoint	Mobile phone-powered AI: 7-class AI and ISIC AI	Specialist and novice clinicians using routine dermoscopic assessment	Histopathology for excised or suspicious lesions	Dermoscopy; AI
Phillips (28)	United Kingdom	Prospective observational clinical validation study	514 patients; 551 biopsied lesions and 999 control lesions imaged	125/426	Deep Ensemble for Recognition of Malignancy algorithm	Specialist clinical assessment	Histopathology for biopsied lesions	Dermoscopy
Winkler (16)	Germany	Prospective two-center diagnostic study	188 patients/228 melanocytic lesions	38/190	Moleanalyzer Pro CNN	Dermatologists alone; dermatologists assisted by CNN	Histopathology for excised lesions; follow-up and expert consensus for non-excised lesions	Dermoscopy; AI; Doctor AI

The main characteristics of the 10 studies included in the main quantitative analysis, including publication year, country or region, study design, sample size, diagnostic method, comparator, reference standard, and included analysis group. CSLM, confocal scanning laser microscopy.

### 3.3 Risk of bias and applicability concerns

**Figure 2:**
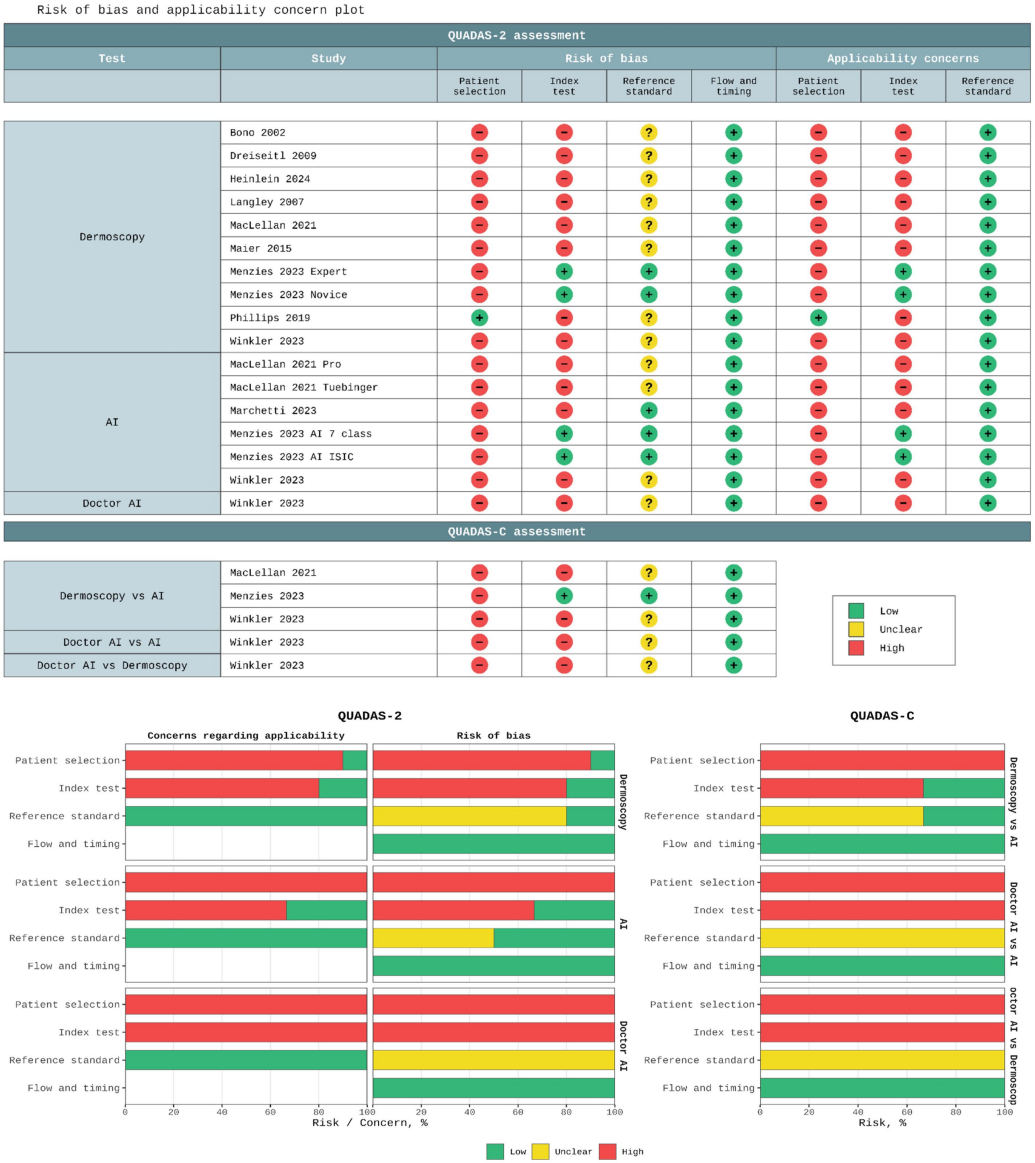
Risk of bias and applicability assessment of the included studies. QUADAS-2 was used to assess the risk of bias and applicability concerns across the included diagnostic arms. QUADAS-C was further used in studies with direct comparisons between dermoscopy, AI, and AI-assisted clinicians. Green indicates low risk, yellow indicates unclear risk, and red indicates high risk.

QUADAS-2 and QUADAS-C were used to assess the methodological quality of the included studies. Overall, the main risk of bias arose from patient selection. The majority of studies included clinically suspicious or biopsy-planned pigmented lesions rather than unselected screening populations. Therefore, patient selection was frequently judged as high risk. For the index test domain, several studies were rated as high risk because of differences in AI algorithms, diagnostic thresholds, clinician experience, and unclear blinding. The reference standard was generally acceptable because the majority of studies used histopathology, although some were rated as unclear when blinding of pathological assessment was not reported. Flow and timing were mostly judged as low risk. Applicability concerns were mainly found in patient selection and index test domains, while concerns about the reference standard were generally low. The detailed QUADAS-2 and QUADAS-C results are shown in Figure 2.

### 3.4 Diagnostic performance and comparative meta-analysis

**Figure 3:**
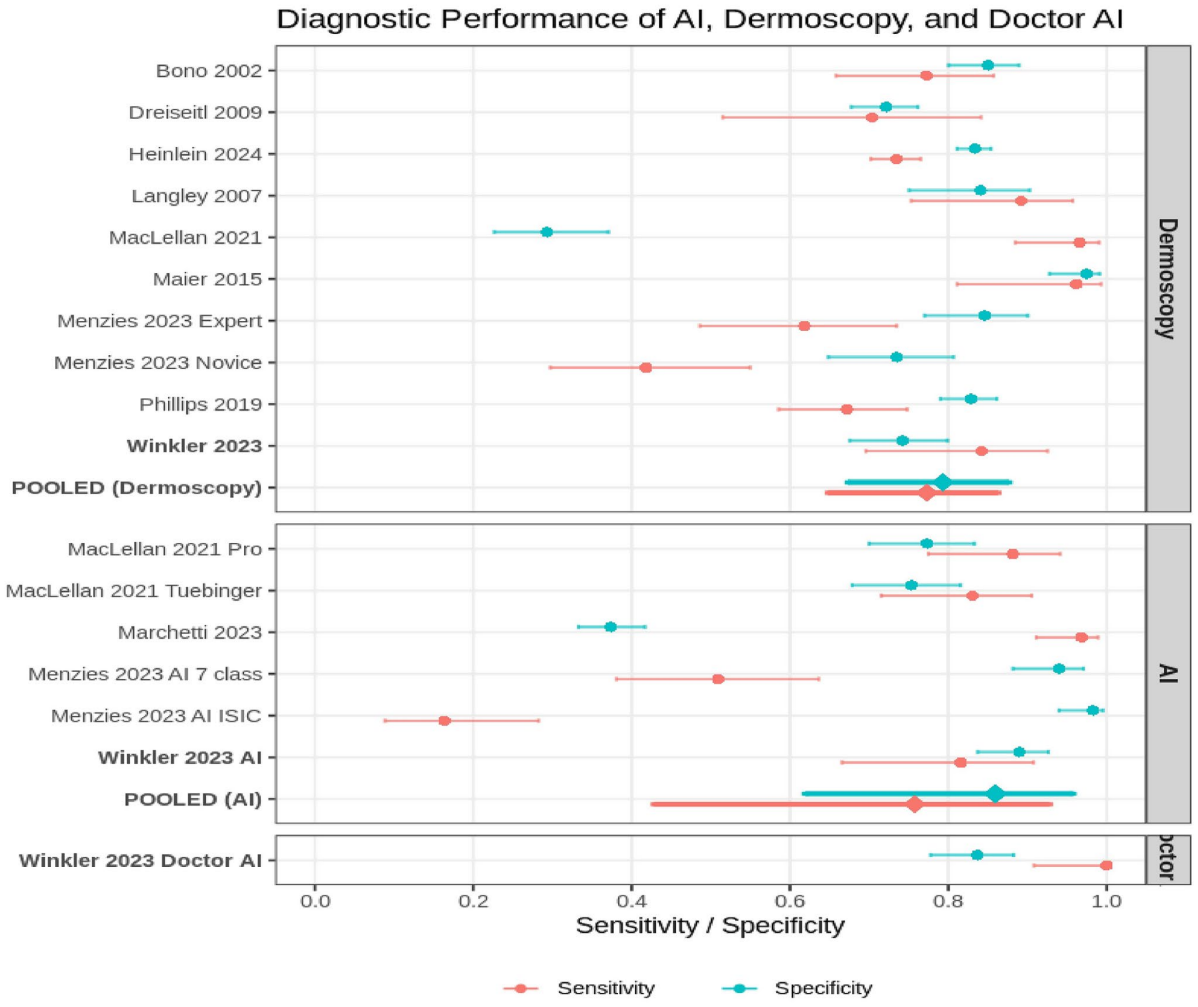
Study-level sensitivity and specificity of AI, dermoscopy, and AI-assisted clinicians. Each point represents the sensitivity or specificity of one diagnostic arm, with horizontal bars indicating 95% confidence intervals. Diagnostic arms were grouped as dermoscopy, AI alone, and AI-assisted clinicians.

Figure 3 summarizes diagnostic accuracy across the study arms. In the dermoscopy group (10 arms), a bivariate random-effects model yielded a pooled sensitivity of 0.773 (95% CI, 0.648–0.863, *p* < 0.001) and a specificity of 0.793 (95% CI, 0.673–0.877, *p* < 0.001). Substantial between-study heterogeneity was observed, with sensitivity ranging from 0.418 to 0.966 and specificity from 0.293 to 0.975, suggesting variation associated with clinician experience and practice settings.

In the standalone artificial intelligence (AI) group (6 arms), pooled sensitivity was 0.757 (95% CI, 0.428–0.928, *p* < 0.001) and specificity was 0.859 (95% CI, 0.619–0.958, *p* < 0.001). A threshold effect was evident across algorithms. The ADAE model favored sensitivity at the expense of specificity, whereas the AI-ISIC arm showed higher specificity with lower sensitivity, indicating differences in model calibration. The performance of AI as an assistive tool was evaluated in Winkler’s study (2023), in which AI-assisted dermatologists achieved a sensitivity of 1.000 and a specificity of 0.837. Although this finding suggests a potential benefit over unaided assessment, the available evidence remains limited and requires prospective validation.

### 3.5 Heterogeneity and threshold effect analysis

Exploration of inter-study variance revealed substantial heterogeneity within both diagnostic modalities. Quantitative assessment using Holling’s *I*^2^ estimates demonstrated moderate to high heterogeneity, ranging from 54.3 to 87.9% in the dermoscopy cohort and 74.7 to 81.2% in the standalone AI cohort. Given the inherent trade-off between sensitivity and specificity in diagnostic test accuracy studies, a bivariate correlation analysis was conducted to ascertain whether this variance was attributable to the threshold effect. The correlation coefficient between logit sensitivity and logit false-positive rate was 0.26 for dermoscopy, indicating that non-threshold factors (e.g., clinician experience, diverse patient demographics, and lesion complexity) primarily drove the observed variability. Conversely, the AI cohort exhibited a perfect positive correlation (*r* = 1.00). This salient finding indicates that the profound heterogeneity observed among AI algorithms is overwhelmingly driven by threshold effects. Specifically, the variance stems from discrepancies in the predefined operating cutoffs optimized by different developers rather than fundamental disparities in the intrinsic discriminatory capacity of the neural networks themselves.

### 3.6 Diagnostic performance metrics

**Figure 4:**
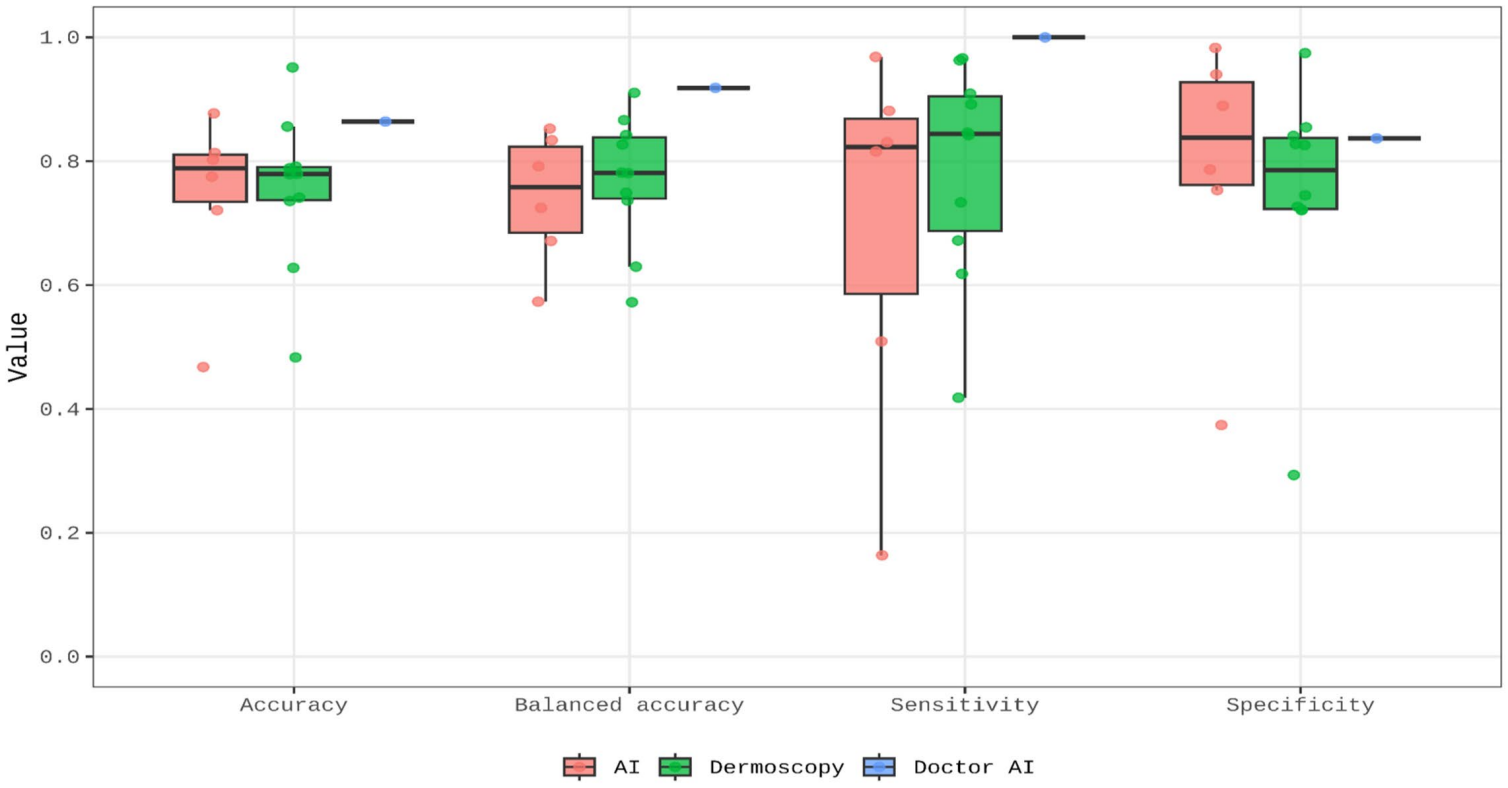
Distribution of diagnostic performance metrics. Box plots show accuracy, balanced accuracy, sensitivity, and specificity across AI, dermoscopy, and AI-assisted clinician groups.

The box plot shows the distribution of accuracy, balanced accuracy, sensitivity, and specificity across the three diagnostic groups. Overall, AI and dermoscopy showed similar distributions in accuracy and balanced accuracy. However, the AI group showed wider variation in sensitivity and specificity, indicating that diagnostic performance differed across AI systems. The dermoscopy group also showed variability, which may be related to differences in clinician experience, diagnostic thresholds, and study populations. The AI-assisted clinician group showed high values across all four metrics in the single available diagnostic arm. However, since this group was represented by only one arm, its distribution cannot be interpreted as a pooled estimate or as comparative evidence against AI-alone or dermoscopy groups. These findings are presented descriptively only. The distribution of diagnostic performance metrics is shown in Figure 4.

### 3.7 Summary ROC analysis

**Figure 5:**
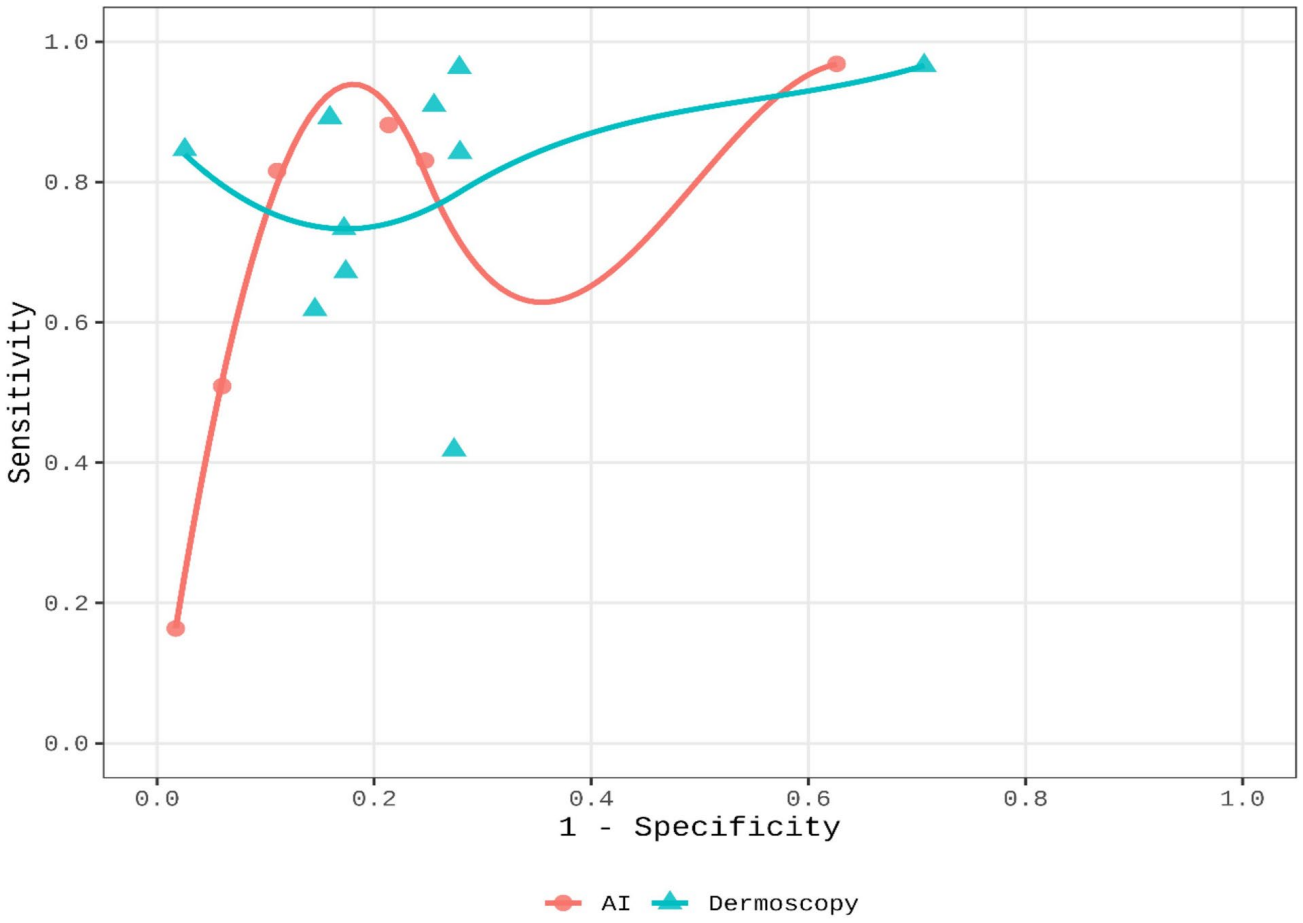
SROC curves of AI and dermoscopy. The SROC plot compares study-level diagnostic performance between AI and dermoscopy using sensitivity and 1 − specificity.

The summary receiver operating characteristic (SROC) plot was used to visually compare the overall diagnostic performance of AI and dermoscopy. The study-level estimates of AI and dermoscopy were distributed in overlapping areas, and no clear separation was observed between the two curves. This suggests that AI alone had an overall diagnostic performance broadly comparable to dermoscopy. However, the distribution of AI points was relatively dispersed, indicating heterogeneity among different AI algorithms and diagnostic thresholds. Similarly, dermoscopy estimates also varied across studies, likely indicating differences in clinician experience and study design. The SROC curves are shown in Figure 5.

### 3.8 Publication bias

**Figure 6:**
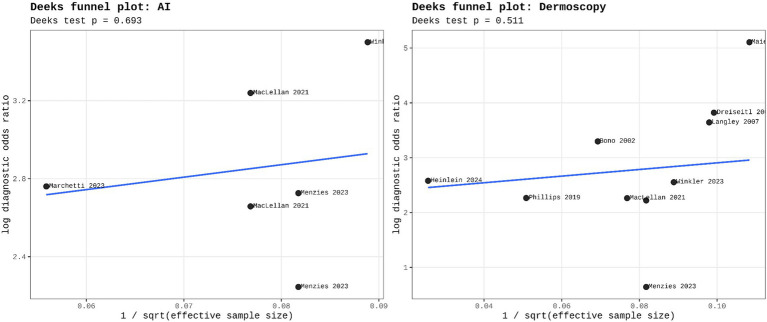
Deeks’ funnel plots for publication bias. Deeks’ funnel plots were used to assess potential publication bias and small-study effects in the AI and dermoscopy groups. No significant publication bias was detected in either the AI group (*p* = 0.693) or the dermoscopy group (*p* = 0.511).

Potential publication bias and small-study effects were assessed using Deeks’ funnel plots. In the AI group, Deeks’s test showed no significant evidence of publication bias (*p* = 0.693). Similarly, no significant publication bias was detected in the dermoscopy group (*p* = 0.511). Visual inspection of the funnel plots also did not suggest obvious asymmetry. However, since the number of included diagnostic arms was limited, especially in the AI group, these results should be interpreted cautiously. Deeks’ funnel plots are shown in Figure 6.

### 3.9 Sensitivity analysis using one arm per study population

Since several studies reported more than one diagnostic arm from the same study population, we performed a sensitivity analysis retaining one clinically primary arm per independent study population within each modality-specific synthesis. Duplicate or secondary arms, including alternative AI thresholds, secondary AI algorithms, novice-reader arms, or secondary teledermoscopy arms, were excluded from this sensitivity analysis according to the prespecified hierarchy. In the one-arm-per-study sensitivity analysis, dermoscopy showed a pooled sensitivity of 0.879 and a specificity of 0.779, whereas standalone AI showed a pooled sensitivity of 0.855 and a specificity of 0.731. Compared with the primary arm-level analysis, point estimates shifted after the removal of secondary reader groups and alternative AI outputs, indicating that correlated arms had some influence on the pooled estimates. However, the overall interpretation remained unchanged: Standalone AI did not show consistent superiority over dermoscopy, and both modalities showed overlapping diagnostic performance. These findings indicate that the main interpretation was not driven solely by double counting of multiple arms from the same study population. Detailed arm-level inclusion and exclusion decisions are presented in Supplementary Table S4, and the one-arm-per-study sensitivity results are shown in Supplementary Table S5.

## 4 Discussion

### 4.1 Principal findings and clinical parity

This systematic review and meta-analysis provides a comprehensive evaluation of the diagnostic efficacy of standalone artificial intelligence (AI), conventional dermoscopy, and AI-assisted clinicians (“Doctor AI”) in the malignancy risk stratification of pigmented skin lesions. Our primary findings indicate that, in prospective, real-world clinical settings, the overall diagnostic performance of autonomous AI systems is broadly comparable to that of standard dermoscopy, as evidenced by the significant overlap in their summary receiver operating characteristic (SROC) curves. Although algorithmic assessment achieved a significantly higher pooled specificity (0.859 vs. 0.793), it was offset by a lower pooled sensitivity (0.757 vs. 0.773). These overlapping performance bounds suggest that, as an independent clinical decision tool, current machine learning models do not yet offer a definitive advantage over the established standard of care.

### 4.2 The “performance gap” between retrospective and prospective evidence

In recent years, deep learning systems, particularly convolutional neural networks (CNNs), have demonstrated transformative potential in dermatological image recognition (29, 30). Numerous retrospective studies using historical image databases have reported that AI can achieve classification accuracies for melanoma that match or even exceed those of experienced board-certified dermatologists (31, 32). However, this analysis corroborates a growing “translation gap”: When these models are transitioned from highly structured experimental datasets to the stochastic nature of prospective clinical practice, their perceived superiority often decreases (33). The high heterogeneity of lesion morphology in the real world, non-standardized lighting and imaging equipment, and the interference of complex, rare pathologies are primary drivers of this performance attrition when translating from “bench to bedside” (34, 35).

These findings should also be interpreted within the broader landscape of assistive imaging technologies for melanoma detection. Techniques such as spectrum-aided vision enhancer (SAVE), hyperspectral or multispectral imaging, reflectance confocal microscopy, and optical coherence tomography may provide complementary information beyond conventional dermoscopy and AI-based classification. Lin et al. have shown that SAVE may enhance the detection of multiple melanoma subtypes by improving spectral contrast compared with white-light imaging. Nevertheless, these technologies differ from the AI and dermoscopy arms included in the present meta-analysis in imaging mechanism, clinical workflow, reference comparison, and reported performance metrics. Therefore, they were used to contextualize emerging diagnostic strategies rather than incorporated into the pooled estimates.

### 4.3 Exploratory threshold effect findings and algorithmic heterogeneity

A discovery of this study is the fundamental difference in the drivers of heterogeneity between the two diagnostic modalities. In the dermoscopy cohort, the correlation coefficients of logit sensitivity and false-positive rates suggest that variance is primarily driven by non-threshold factors, such as clinician expertise, training background, and diverse patient demographics. Conversely, the standalone AI group exhibited a perfect positive correlation (*r* = 1.00), indicating that the observed heterogeneity is almost exclusively attributable to “threshold effects.” This finding suggests that the performance variance across different AI systems reflects developers’ distinct calibration strategies for operating cutoffs, prioritizing high sensitivity to minimize missed diagnoses and high specificity to reduce unnecessary biopsies rather than fundamental disparities in the intrinsic discriminatory capacity of the neural networks themselves. Consequently, there is an urgent need for precise calibration of AI probability outputs and the establishment of standardized clinical operating thresholds (36).

### 4.4 Dataset bias and medical equity

The limitations of AI performance should also be viewed through the lens of dataset representational bias. Current mainstream models rely heavily on a few large-scale, open-source repositories, such as the HAM10000 and ISIC archives (37, 38). Evidence has suggested that dark skin tones (Fitzpatrick types IV–VI) are significantly underrepresented in these datasets, resulting in a significant decrease in generalizability and a risk of algorithmic bias when applied to diverse global populations (39, 40). Furthermore, isolated image-based inputs deprive the models of critical clinical metadata such as patient history, rate of lesion evolution, and personal or family history of skin cancer, which are essential for definitive diagnosis (41). The absence of this clinical context likely imposes a “ceiling effect” on the diagnostic accuracy of standalone image-analysis AI.

### 4.5 Preliminary evidence on AI-assisted clinicians

Given the limitations of standalone AI, AI-assisted clinical assessment remains an important area for future investigation. In this review, only one diagnostic arm evaluated dermatologists assisted by AI, reporting a sensitivity of 1.000 and a specificity of 0.837. Although this result suggests that human–AI collaboration may be worth further study, the evidence is insufficient to support a practical recommendation or to conclude that AI assistance can reliably reduce experience-related disparities in clinical decision-making.

Accordingly, the AI-assisted clinician finding should be regarded as hypothesis-generating. Future research should evaluate whether AI support improves diagnostic accuracy, calibration, referral decisions, biopsy decisions, clinician confidence, and patient-centered outcomes under standardized clinical workflows. Such studies should ensure consistent physician expertise across comparison arms, prespecified AI thresholds, transparent reporting of algorithm outputs, and adequate representation of diverse patient populations and lesion types.

### 4.6 Emerging adjunctive technologies beyond AI and dermoscopy

Melanoma risk assessment should also be considered within a broader methodological framework that includes adjunctive visual enhancement and non-invasive imaging technologies. These approaches, such as spectrum-aided vision enhancement, multispectral or hyperspectral imaging, reflectance confocal microscopy, and optical coherence tomography, may provide complementary information beyond conventional dermoscopy or AI-based image classification. Lin et al. have shown that the spectrum-aided vision enhancer (SAVE) may improve visualization of multiple melanoma subtypes, including acral lentiginous, nodular, *in situ*, and superficial spreading melanoma (42). However, SAVE differs from dermoscopy-based AI in imaging mechanism, clinical workflow, diagnostic threshold, and reported outcomes. Future studies should standardize reference standards and report extractable 2 × 2 diagnostic data to support comparative synthesis.

### 4.7 Strengths and limitations

This study strictly focused on prospective clinical trials, effectively filtering out the “laboratory bias” inherent in pure algorithm development studies. However, several limitations remain. First, the majority of the included studies were judged as high risk for patient selection bias, as they included patients already identified with suspicious lesions rather than unselected screening populations. Second, the diversity of AI architectures and the relative scarcity of head-to-head “Doctor AI” trials limit our ability to draw definitive conclusions about the optimal integration strategy.

## 5 Conclusion

This systematic review and meta-analysis indicates that standalone AI demonstrates diagnostic performance broadly comparable to conventional dermoscopy for the malignancy risk stratification of pigmented skin lesions; however, current evidence does not substantiate the consistent superiority of autonomous AI. The marked variability across AI systems underscores that algorithmic efficacy remains highly context-dependent. Evidence on AI-assisted clinical assessment remains limited to a single diagnostic arm and should therefore be regarded as preliminary and hypothesis-generating. At present, AI should be conceptualized not as a replacement for dermatological expertise but as a supplementary tool to optimize diagnostic decision-making. Before AI can be responsibly evaluated for integration into routine skin lesion triage pathways, future research must prioritize prospective multicenter designs, diverse patient cohorts, transparent algorithmic reporting, standardized operating thresholds, and clinically significant patient-centered outcomes.

## Data Availability

The original contributions presented in the study are included in the article/Supplementary material, further inquiries can be directed to the corresponding author.
