# Supplementary material for: Artificial intelligence vs. dermoscopy for malignancy risk stratification of pigmented skin lesions: a systematic review and meta-analysis for public health
Source: Front Med (Lausanne). 2026 Jul 28;13:1883944. doi: 10.3389/fmed.2026.1883944 (PMC13457125; doi:10.3389/fmed.2026.1883944)
Supplement: Supplementary file 2 [file Table_2.docx]

**Table S2**. Electronic Database Search Strategies for the Systematic Review and Meta-analysis

| **Database** | **Search Period** | **Search Strategy** |
| --- | --- | --- |
| PubMed | From database inception to January 2026 | ("Artificial Intelligence"[Mesh] OR "Machine Learning"[Mesh] OR "Deep Learning"[Mesh] OR artificial intelligence[tiab] OR AI[tiab] OR machine learning[tiab] OR deep learning[tiab] OR neural network*[tiab] OR convolutional neural network*[tiab] OR CNN[tiab] OR computer-assisted diagnosis[tiab] OR automated diagnosis[tiab] OR algorithm*[tiab] OR diagnostic algorithm*[tiab])  AND  ("Dermoscopy"[Mesh] OR dermoscopy[tiab] OR dermatoscopy[tiab] OR epiluminescence microscopy[tiab] OR digital dermoscopy[tiab] OR dermoscopic[tiab])  AND  ("Skin Neoplasms"[Mesh] OR "Melanoma"[Mesh] OR pigmented lesion*[tiab] OR pigmented skin lesion*[tiab] OR melanocytic lesion*[tiab] OR melanocytic nev*[tiab] OR melanoma*[tiab] OR skin cancer*[tiab] OR cutaneous melanoma[tiab] OR atypical nevus[tiab] OR dysplastic nevus[tiab])  AND  (diagnostic accuracy[tiab] OR sensitivity[tiab] OR specificity[tiab] OR ROC[tiab] OR AUC[tiab] OR receiver operating characteristic[tiab] OR predictive value*[tiab] OR diagnostic performance[tiab] OR risk stratification[tiab])  AND  (skin of color[tiab] OR darker skin[tiab] OR dark skin[tiab] OR Fitzpatrick[tiab] OR Fitzpatrick type IV[tiab] OR Fitzpatrick type V[tiab] OR Fitzpatrick type VI[tiab] OR ethnic skin[tiab] OR highly pigmented skin[tiab])  NOT  (animal[mh] NOT human[mh]) |
| Embase | From database inception to January 2026 | ('exp artificial intelligence'/de OR 'exp machine learning'/de OR 'exp deep learning'/de OR 'artificial intelligence':ti,ab OR 'machine learning':ti,ab OR 'deep learning':ti,ab OR 'neural network*':ti,ab OR 'convolutional neural network*':ti,ab OR cnn:ti,ab OR 'computer assisted diagnosis':ti,ab)  AND  ('exp dermoscopy'/de OR dermoscopy:ti,ab OR dermatoscopy:ti,ab OR dermoscopic:ti,ab OR 'epiluminescence microscopy':ti,ab)  AND  ('exp melanoma'/de OR 'exp skin tumor'/de OR 'pigmented lesion*':ti,ab OR 'pigmented skin lesion*':ti,ab OR melanoma*:ti,ab OR 'melanocytic lesion*':ti,ab OR 'skin cancer*':ti,ab)  AND  ('diagnostic accuracy':ti,ab OR sensitivity:ti,ab OR specificity:ti,ab OR 'diagnostic performance':ti,ab OR 'risk stratification':ti,ab OR auc:ti,ab OR roc:ti,ab)  AND  ('skin of color':ti,ab OR 'dark skin':ti,ab OR Fitzpatrick:ti,ab OR 'ethnic skin':ti,ab) |
| Web of Science (WoS) | From database inception to January 2026 | TS=(  ("artificial intelligence" OR "machine learning" OR "deep learning" OR "neural network*" OR "convolutional neural network*" OR "computer-assisted diagnosis" OR "automated diagnosis")  AND  (dermoscopy OR dermatoscopy OR dermoscopic OR "epiluminescence microscopy")  AND  ("pigmented lesion*" OR "pigmented skin lesion*" OR melanoma* OR "melanocytic lesion*" OR "skin cancer*" OR "cutaneous melanoma")  AND  ("diagnostic accuracy" OR sensitivity OR specificity OR ROC OR AUC OR "diagnostic performance" OR "risk stratification")  AND  ("skin of color" OR "dark skin" OR Fitzpatrick OR "ethnic skin")  ) |
| Cochrane Library | From database inception to January 2026 | ([mh "Artificial Intelligence"] OR [mh "Machine Learning"] OR [mh "Deep Learning"] OR "artificial intelligence":ti,ab,kw OR "machine learning":ti,ab,kw OR "deep learning":ti,ab,kw OR "neural network*":ti,ab,kw OR "convolutional neural network*":ti,ab,kw OR cnn:ti,ab,kw OR "computer assisted diagnosis":ti,ab,kw)  AND  ([mh Dermoscopy] OR dermoscopy:ti,ab,kw OR dermatoscopy:ti,ab,kw OR dermoscopic:ti,ab,kw OR "epiluminescence microscopy":ti,ab,kw)  AND  ([mh Melanoma] OR [mh "Skin Neoplasms"] OR "pigmented lesion*":ti,ab,kw OR "pigmented skin lesion*":ti,ab,kw OR melanoma*:ti,ab,kw OR "melanocytic lesion*":ti,ab,kw OR "skin cancer*":ti,ab,kw)  AND  ("diagnostic accuracy":ti,ab,kw OR sensitivity:ti,ab,kw OR specificity:ti,ab,kw OR "diagnostic performance":ti,ab,kw OR "risk stratification":ti,ab,kw OR auc:ti,ab,kw OR roc:ti,ab,kw)  AND  ("skin of color":ti,ab,kw OR "dark skin":ti,ab,kw OR Fitzpatrick:ti,ab,kw OR "ethnic skin":ti,ab,kw) |

Table Legend

This table presents the complete electronic search strategies used in four major biomedical databases, developed in strict accordance with the PRISMA-DTA (Preferred Reporting Items for Systematic Reviews and Meta-Analyses of Diagnostic Test Accuracy) guidelines. All searches were conducted in January 2026, covering all literature from database inception to the search date.

**Field abbreviations:**

ti: Title

ab: Abstract

kw: Keyword

mh: Medical Subject Headings (MeSH, used in PubMed and Cochrane Library)

de: Emtree descriptor (used in Embase)

TS: Topic search (used in Web of Science, covering title, abstract, and keywords)

**Notes:**

MeSH terms and Emtree terms were used to maximize the sensitivity of subject-based searches

Wildcard character (*) was used to capture all variant forms of keywords

Non-human animal studies were explicitly excluded from the PubMed search

All search strategies were independently verified by two reviewers to ensure accuracy and completeness
