# Supplementary material for: Artificial intelligence vs. dermoscopy for malignancy risk stratification of pigmented skin lesions: a systematic review and meta-analysis for public health
Source: Front Med (Lausanne). 2026 Jul 28;13:1883944. doi: 10.3389/fmed.2026.1883944 (PMC13457125; doi:10.3389/fmed.2026.1883944)
Supplement: Supplementary file 3 [file Table_3.docx]

**Table S3.** Standardized Data Extraction Form Template and Reconstructed 2×2 Contingency Tables for Diagnostic Accuracy Studies of Artificial Intelligence, Dermoscopy, and AI-Assisted Clinicians in Pigmented Skin Lesion Malignancy Risk Stratification

| **Study ID** | **Diagnostic Arm Type** | **Diagnostic Method/AI System** | **Total Lesions** | **Malignant Lesions** | **Benign Lesions** | **TP** | **FP** | **FN** | **TN** | **Sensitivity** | **Specificity** | **Data Source** |
| --- | --- | --- | --- | --- | --- | --- | --- | --- | --- | --- | --- | --- |
| **Bono 2002** | Dermoscopy | Clinician dermoscopy | 313 | 66 | 247 | 60 | 63 | 6 | 184 | 0.909 | 0.745 | Directly extracted |
| **Dreiseitl 2009** | Dermoscopy | Expert dermoscopy | 458 | 27 | 431 | 26 | 120 | 1 | 311 | 0.963 | 0.722 | Directly extracted |
| **Heinlein 2024** | Dermoscopy | Dermatologist dermoscopy | 1571 | 750 | 821 | 479 | 142 | 174 | 776 | 0.639 | 0.845 | Directly extracted |
| **Langley 2007** | Dermoscopy | Clinician dermoscopy | 125 | 37 | 88 | 33 | 14 | 4 | 74 | 0.892 | 0.841 | Directly extracted |
| **MacLellan 2021** | Dermoscopy | Local dermatologist dermoscopy | 209 | 59 | 150 | 57 | 102 | 2 | 48 | 0.966 | 0.320 | Directly extracted |
| **MacLellan 2021** | Dermoscopy | Teledermoscopy | 209 | 59 | 150 | 55 | 106 | 4 | 44 | 0.932 | 0.293 | Reconstructed |
| **Maier 2015** | Dermoscopy | Dermatologist dermoscopy | 144 | 26 | 118 | 22 | 3 | 4 | 115 | 0.846 | 0.975 | Directly extracted |
| **Menzies 2023** | Dermoscopy | Expert dermoscopy | 172 | 55 | 117 | 62 | 12 | 22 | 76 | 0.738 | 0.863 | Directly extracted |
| **Menzies 2023** | Dermoscopy | Novice dermoscopy | 172 | 55 | 117 | 23 | 10 | 32 | 107 | 0.418 | 0.915 | Reconstructed |
| **Phillips 2019** | Dermoscopy | Specialist clinical assessment | 551 | 125 | 426 | 119 | 104 | 6 | 322 | 0.952 | 0.756 | Directly extracted |
| **Winkler 2023** | Dermoscopy | Dermatologist dermoscopy | 228 | 38 | 190 | 32 | 53 | 6 | 137 | 0.842 | 0.721 | Directly extracted |
| **MacLellan 2021** | AI alone | FotoFinder Moleanalyzer Pro | 209 | 59 | 150 | 52 | 32 | 7 | 118 | 0.881 | 0.787 | Directly extracted |
| **MacLellan 2021** | AI alone | FotoFinder Tuebinger | 209 | 59 | 150 | 48 | 28 | 11 | 122 | 0.814 | 0.813 | Reconstructed |
| **Marchetti 2023** | AI alone | ADAE open-source AI algorithm | 603 | 95 | 508 | 92 | 318 | 3 | 190 | 0.968 | 0.374 | Directly extracted |
| **Menzies 2023** | AI alone | 7-class AI | 172 | 55 | 117 | 53 | 21 | 31 | 67 | 0.631 | 0.761 | Directly extracted |
| **Menzies 2023** | AI alone | ISIC AI | 172 | 55 | 117 | 9 | 2 | 46 | 115 | 0.164 | 0.983 | Reconstructed |
| **Winkler 2023** | AI alone | Moleanalyzer Pro CNN | 228 | 38 | 190 | 31 | 21 | 7 | 169 | 0.816 | 0.889 | Directly extracted |
| **Winkler 2023** | AI-assisted clinician | Dermatologist + Moleanalyzer Pro | 228 | 38 | 190 | 38 | 31 | 0 | 159 | 1.000 | 0.837 | Directly extracted |

**Notes**

1． All data marked as "Directly extracted" were obtained from explicitly reported 2×2 contingency tables or TP/FP/FN/TN values in the original studies.
2． Data marked as "Reconstructed" were calculated using the following formulas based on reported sensitivity, specificity, and total number of lesions:

TP = Sensitivity × Number of malignant lesions

TN = Specificity × Number of benign lesions

FP = Number of benign lesions − TN

FN = Number of malignant lesions − TP

3． All diagnostic counts are rounded to integers; sensitivity and specificity are reported to three decimal places.
4． This table strictly corresponds to the 17 diagnostic arms included in the main meta-analysis and excludes data from supplementary analysis studies.
