# Supplementary material for: Artificial intelligence vs. dermoscopy for malignancy risk stratification of pigmented skin lesions: a systematic review and meta-analysis for public health
Source: Front Med (Lausanne). 2026 Jul 28;13:1883944. doi: 10.3389/fmed.2026.1883944 (PMC13457125; doi:10.3389/fmed.2026.1883944)
Supplement: Supplementary file 4 [file Table_4.docx]

**Table S4** Arm inclusion matrix

| **Study** | **Arm** | **Modality** | **Included in main arm-level analysis?** | **Included in one-arm sensitivity analysis?** | **Reason** |
| --- | --- | --- | --- | --- | --- |
| MacLellan 2021 | Local dermatologist dermoscopy | Dermoscopy | Yes | Yes | Clinically primary dermatologist assessment |
| MacLellan 2021 | Teledermoscopy | Dermoscopy | Yes/Exploratory | No | Secondary reader/setting from same population |
| MacLellan 2021 | FotoFinder Moleanalyzer Pro | AI | Yes | Yes | Primary/commercially intended AI arm |
| MacLellan 2021 | FotoFinder Tuebinger | AI | Yes/Exploratory | No | Alternative AI algorithm from same population |
| Menzies 2023 | Expert dermoscopy | Dermoscopy | Yes | Yes | Expert clinical comparator |
| Menzies 2023 | Novice dermoscopy | Dermoscopy | Yes/Exploratory | No | Secondary reader-experience arm |
| Menzies 2023 | 7-class AI | AI | Yes | Yes | Primary AI diagnostic arm |
| Menzies 2023 | ISIC AI | AI | Yes/Exploratory | No | Alternative AI threshold/model from same population |
| Winkler 2023 | Dermoscopy | Dermoscopy | Yes | Yes | Clinical comparator |
| Winkler 2023 | AI alone | AI | Yes | Yes | Standalone AI comparator |
| Winkler 2023 | AI-assisted clinician | AI-assisted | Yes | Not pooled | Single available AI-assisted arm |
