# Supplementary material for: Artificial intelligence vs. dermoscopy for malignancy risk stratification of pigmented skin lesions: a systematic review and meta-analysis for public health
Source: Front Med (Lausanne). 2026 Jul 28;13:1883944. doi: 10.3389/fmed.2026.1883944 (PMC13457125; doi:10.3389/fmed.2026.1883944)
Supplement: Supplementary file 5 [file Table_5.docx]

**Table S5**. Sensitivity analysis using one diagnostic arm per independent study population

| Analysis | Diagnostic modality | Included studies/arms | Pooled sensitivity (95% CI) | Pooled specificity (95% CI) | Duplicate or secondary arms excluded | Interpretation |
| --- | --- | --- | --- | --- | --- | --- |
| Primary arm-level analysis | Dermoscopy | 10 diagnostic arms | 0.773 (0.648–0.863) | 0.793 (0.673–0.877) | Not applicable | Main exploratory arm-level estimate. |
| Primary arm-level analysis | AI alone | 6 diagnostic arms | 0.757  (0.428–0.928) | 0.859  (0.619–0.958) | Not applicable | Main exploratory arm-level estimate. |
| Primary arm-level analysis | AI-assisted clinician | 1 diagnostic arm | 1.000 | 0.837 | Not applicable | Not pooled because only one eligible diagnostic arm was available. |
| One-arm-per-study sensitivity analysis | Dermoscopy | 9 independent study populations: Bono 2002; Dreiseitl 2009; Heinlein 2024; Langley 2007; MacLellan 2021 local dermatologist dermoscopy; Maier 2015; Menzies 2023 expert dermoscopy; Phillips 2019; Winkler 2023 dermatologist dermoscopy | 0.879  (0.798–0.930) | 0.779  (0.656–0.867) | MacLellan 2021 teledermoscopy; Menzies 2023 novice dermoscopy | Results remained broadly consistent with the primary analysis. Removing secondary reader/setting arms did not materially change the conclusion that dermoscopy showed variable but clinically comparable performance. |
| One-arm-per-study sensitivity analysis | AI alone | 4 independent study populations: MacLellan 2021 FotoFinder Moleanalyzer Pro; Marchetti 2023 ADAE; Menzies 2023 7-class AI; Winkler 2023 Moleanalyzer Pro CNN | 0.855  (0.667–0.946) | 0.731  (0.515–0.874) | MacLellan 2021 FotoFinder Tuebinger; Menzies 2023 ISIC AI | Results remained directionally consistent with the main conclusion. After removing alternative AI models or thresholds from the same study population, standalone AI did not show consistent superiority over dermoscopy. |
| One-arm-per-study sensitivity analysis | AI-assisted clinician | 1 independent study population: Winkler 2023 dermatologist + Moleanalyzer Pro | 1.000  (0.907–1.000) | 0.837  (0.776–0.886) | None | Not pooled because only one AI-assisted clinician arm was available. The estimate should be interpreted cautiously. |
